# Comprehensive Evaluation of White Matter Damage and Neuron Death and Whole-Transcriptome Analysis of Rats With Chronic Cerebral Hypoperfusion

**DOI:** 10.3389/fncel.2019.00310

**Published:** 2019-07-17

**Authors:** Wenxian Li, Di Wei, Jianye Liang, Xiaomei Xie, Kangping Song, Li’an Huang

**Affiliations:** ^1^Department of Neurology, The First Affiliated Hospital, Jinan University, Guangzhou, China; ^2^Department of Neurology, The Second Affiliated Hospital, Xi’an Jiaotong University, Xi’an, China; ^3^Department of Urology, Xijing Hospital, The Fourth Military Medical University, Xi’an, China; ^4^Medical Imaging Center, The First Affiliated Hospital, Jinan University, Guangzhou, China

**Keywords:** chronic cerebral hypoperfusion, white matter damage, neuron death, whole-transcriptome, vascular dementia

## Abstract

**Background/Aims:**

Chronic cerebral hypoperfusion (CCH) is induced by chronic deficit of brain perfusion, contributes to a persistent or progressive cognitive dysfunction, which is characterized by diverse neuropathological manifestations. There are currently no effective medications available. White matter damage (WMD) and cortical neuron death may be caused by CCH, which are related to cognitive impairment, while the underlying molecular mechanisms remain unclear. In the study, a database of the transcriptome level was built to determine potential biomarkers in cortex of CCH.

**Methods:**

CCH was induced in male Sprague-Dawley rats by permanent occlusion of the bilateral common carotid arteries. Rats were randomly divided into three groups: Sham-operated group (*n* = 24), the 4th and 8th week of CCH groups (total = 56, *n* = 28 for each group). Cognitive function was evaluated using the Morris water maze task. WMD and neuron damage were detected using diffusion tensor imaging and histological analysis, respectively. Western blotting analysis of various markers was used to examine neuronal death. Whole-transcriptome microarray was performed to assess mRNA, circRNA, and lncRNA expression profiles at 4th and 8th weeks after CCH. Diversified bioinformatic tools were performed to analyze and predict the key biological processes and signaling pathways of differentially expressed RNAs and co-expressed potential target genes. Co-expression networks of mRNA–circRNA–miRNA and lncRNA–mRNA were constructed.

**Results:**

Compared to the sham group, cognitive impairment, disintegration of white matter, blood-brain barrier damage and neuron death were induced by CCH. Neuron death including apoptosis and necroptosis might occur in the cortex of CCH. We constructed the regulatory networks of whole-transcriptomic including differentially expressed mRNAs, circRNAs, and lncRNAs, and related biological functions and pathways involved in neurological disease, cell death and survival, energy and metabolism, et al. Our results also indicated that Cyr61 mRNA may play a role in the CCH-related cortical neuronal death.

**Conclusion:**

WMD and cortical neuronal death are worthy of attention in the pathogenesis of CCH. Additionally, the present results provide potential evidence at the whole-transcription level for CCH, offering candidate biomarkers and therapeutic targets.

## Introduction

Chronic cerebral hypoperfusion (CCH) is induced by moderate and persistent decrease of cerebral blood flow (CBF). Such pattern of hypoperfusion results in the development and progression of cognitive impairment, such as vascular dementia (VD) (Du et al., 2017). Clinical researches suggested that cerebral hypoperfusion precedes and possibly contributes to onset of dementia (Ruitenberg et al., 2005). And in the general population, cerebral hypoperfusion is associated with accelerated cognitive decline and an increased risk of dementia (Wolters et al., 2017). The subtypes of clinical VD also explicitly includes hypoperfusion dementia (O’Brien and Thomas, 2015). Vascular cognitive deficits are secondary to any type of cerebrovascular disease, the deficit brain perfusions have been identified and raised (Sun et al., 2016). Different from the narrow time window of acute cerebral ischemia, CCH/chronic cerebral ischemia is an available state that can be regulated. CCH experimental model is an effective tool to study the pathological condition, process of degeneration, and mechanisms underlying ischemic cognitive impairment (Du et al., 2017). A minimally invasive bilateral common carotid arterial occlusion (BCCAO) surgery has been used to develop a model of CCH (Zhang T. et al., 2017; Zou et al., 2018; Mao et al., 2019).

Most known mechanisms of CCH include neuroinflammation, oxidative stress injury, dysfunction of neurotransmitter system and mitochondria, disturbance of lipid metabolism, and alterations of growth factors (Du et al., 2017). Neuronal cell death is hallmark of VD induced by CCH (Chen et al., 2017; Mao et al., 2019). Except for the loss of hippocampal neurons, neuron death/neurodegeneration in cortex is another characteristic in CCH brain due to long-term hypoperfusion attack. In addition, white matter damage (WMD) has been frequently detected by imaging scans in clinical subcortical ischemic VD patients induced by CCH. Cerebral WMD contains the loss of oligodendrocyte and demyelination, which takes place after CCH and leads to axonal degeneration (Ma et al., 2018). Therefore, neuron death in cortex may be related to WMD and cognitive impairment directly. However, we know little about the complex pathogenesis of CCH, particularly at the molecular level.

Human genome-wide association studies have demonstrated that a large number of disease-associated genomic variations exist outside the protein-coding genes (Gopalakrishnan et al., 2015). Transcriptomic analyses such as mRNAs, circRNAs, and lncRNAs can provide comprehensive, complete, and specific information relating to a given tissue at a specific time, which bring many advantages including the discovery of novel genomic sequences and the accurate quantification of expression levels. These advantages allow us to create an in-depth understanding of molecular mechanisms and therefore make it possible to identify innovative therapeutic targets (Sun et al., 2019). However, comprehensive analyses of differentially expressed profiles of the whole transcriptome for diseases of the nervous system have not yet been investigated.

In this study, we examined expression profiles and related networks of mRNAs, circRNAs and lncRNAs, in the cortical tissue of a CCH rat model. Gene Ontology (GO), Kyoto Encyclopedia of Genes and Genomes (KEGG), and Reactome enrichment analyses and Ingenuity Pathway Analysis (IPA) were performed in this study. Co-expression and networks of potential targeting relationship were constructed. In addition, diffusion tensor imaging (DTI) was used to specifically investigate the disintegration of white matter, and various methods to examine cell death. The present study has provided new insights into the molecular mechanisms of CCH using whole transcriptome sequencing and bioinformatic approaches.

## Materials and Methods

### Ethics Statement

All animal work was performed in accordance with the Chinese Animal Welfare Act and all experiments involving animals were approved by the Laboratory Animal Ethics Committee of Jinan University (Approval No. IACUC-20180130-03).

### Animals and Surgery

Sprague-Dawley rats were purchased from Laboratory Animal Center, Southern Medical University [Quality certificate No. SYXK(guangdong) 2017-0174, Guangzhou, China]. Eight-week-old male Sprague Dawley rats (weighed between 350 and 400 g) were specific pathogen free. We kept individually in a vivarium of constant temperature (25 ± 1°C) and 40–70% humidity and exposed them to unlimited food and water. We also exposed them to alternating 12:12-h light and dark cycle (light cycle: from 8:00 am to 8:00 pm; dark cycle: from 8:00 pm to 8:00 am) for 4 weeks of adaptive feeding, before all the animal experiments. Precautions were taken to keep the number of animals used to a low and to mitigate pain.

A total of 80 rats were used and randomized in this study. BCCAO operation was used to induce CCH in rats. Each rat was anesthetized with an intraperitoneal injection of 3% pentobarbital sodium salt (0.2 mL/kg body weight). A median incision was made in the rat’s neck, and the bilateral common carotid arteries and the vagus nerves were then separated and isolated. In the BCCAO rats, the bilateral carotid arteries were double-ligated with 2-0 sutures. The BCCAO groups, or the CCH groups, included two subgroups: the 4th week of CCH group (CCH 4W; *n* = 28) and the 8th week of CCH group (CCH 8W; *n* = 28). In the sham-operated group (*n* = 24), the bilateral carotid arteries were separated as did in the BCCAO rats but were not ligated. The wounds were then closed. The body temperature of the rats was maintained at 37 ± 2°C using a heating lamp during surgery as well as during recovery from anesthesia.

### Morris Water Maze Task

The cognitive function was evaluated using the Morris water maze task (MWT) (Ethovison XT, Noldus Information Technology Co., Hague, Netherlands). A total of 18 rats (Sham, *n* = 6; CCH 4W, *n* = 6; CCH 8W, *n* = 6) were assessed. The test was performed according to our previously described protocol (Jing et al., 2015; Xiong et al., 2017).

### Magnetic Resonance Imaging

To evaluate white matter integrity, magnetic resonance imaging (MRI) with DTI detection was conducted using a Discovery 750 3.0T scanner (an 8-channel wrist coil, GE Healthcare, Milwaukee, WI, United States). The corpus callosum (CC) and both external capsules (ECs), where white matter tracts are the most abundant in the rat brain, were selected for T2-weighted imaging (T2WI; Figure 4A). Scanning methods according to a previous protocol, and data acquisition was performed using parameters as described previously (Jing et al., 2015; Choi et al., 2016; Xiong et al., 2017). The DTI parameter maps of fractional anisotropy (FA) were calculated.

Rats in the vehicle group (*n* = 6) were scanned using MRI at three time points: pre-occlusion and 4th and 8th weeks after BCCAO (vehicle).

### Evaluation of Neuronal Damage Using Hematoxylin and Eosin and Nissl Staining

The rats were euthanized, and the rat-brain samples were collected and fixed in 4% paraformaldehyde for 48 h; subsequently, these brain samples were embedded in paraffin and sectioned at 5-μm intervals. To evaluate neuron damage, hematoxylin and eosin (HE) and Nissl staining were carried out according to the manufacturer’s instructions. The mounted slides were then examined and photographed using a Nikon^®^ DS-U3 imaging system (Japan).

### Fluoro-Jade B Histofluorescence

To investigate neurodegeneration, sham- and BCCAO-operated animals (three rats in each group were assessed for statistical analysis) were used for Fluoro-Jade B (FJB) (Millipore) histofluorescence analysis. FJB and DAPI (Sigma) staining were performed according to a previous protocol (Caccamo et al., 2017). All images were acquired using a Nikon^®^ C2 confocal microscope (Japan) under 200 magnification.

### Immunohistochemical Staining

Immunohistochemical staining was performed as described previously (Kiyota et al., 2018). For Aβ_1–42_ immunostaining (Aβ_1–42_ antibody, 1:100; Abcam), Image J was used to measure intracellular Aβ_1–42_-positive cells and the total areas of plaques after adjusting for the threshold. Intracellular Aβ_1–42_-positive cells and extracellular Aβ_1–42_ plaques and were measured, respectively. Three rats in each group were assessed for statistical analysis.

To confirm WMD, diaminobenzidine staining for myelin basic protein (MBP; 1:100, Abcam) and luxol fast blue (LFB; Sigma) stainings were performed as previously described (Choi et al., 2016; Ghaiad et al., 2017) to examine WMD. The WMD was evaluated in the CC and both ECs. In the MBP staining, normal myelination was scored 3 (Choi et al., 2016), and in the LFB staining, demyelination was scored from 0 to 3. A score of 0 refers to a normal myelin status; a score of 1 indicates demyelination of one-third of the myelin tract fibers; a score of 2 indicates demyelination of two-thirds of the myelin tract fibers; a score of 3 refers to complete demyelination(Ghaiad et al., 2017). Scores from different sections were summed up to obtain an average score for each group. Data were pooled from 3 sections at 200 magnifications for each rat, and 3 rats in each group were assessed for statistical analysis.

### RNA Isolation

Total RNA was isolated from cerebral cortex tissues using HiPure Total RNA Mini Kit (Magen) according to the manufacturer’s protocol. The concentration and integrity of the extracted total RNA were estimated by Qubit 3.0 Fluorometer (Invitrogen) and Agilent 2100 Bioanalyzer (Applied Biosystems), respectively. RNA samples with an RIN value of at least 7.0 or higher were used for further processing.

### Library Preparation for RNA Sequencing

**FIGURE 1:**
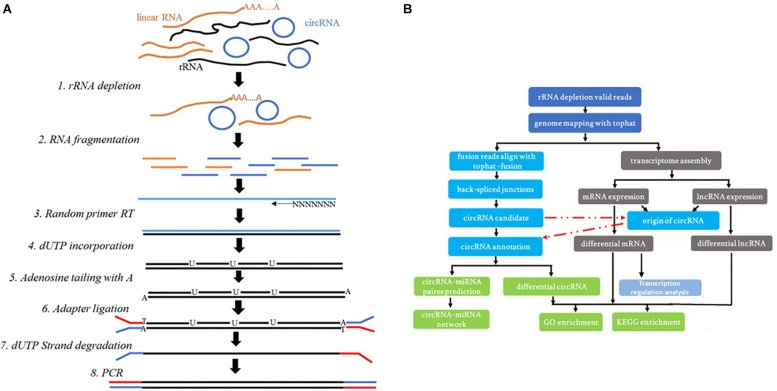
**(A)** The experimental technical process. **(B)** The technical process of the whole-transcriptome analysis.

An RNA-seq library was prepared with approximately 1 μg of total RNA using a KAPA RNA HyperPrep Kit with RiboErase (HMR) for Illumina^®^ (Kapa Biosystems). Briefly, ribosomal RNA was removed from the total RNA. Next, the ribominus RNA was fragmented, and the first strand and directional second strand synthesis was performed. A tailing and adapter ligation were subsequently performed using the purified cDNA. Finally, the purified, adapter-ligated DNA was amplified. The DNA library quality and concentration were assessed using a DNA 1000 chip on an Agilent 2100 Bioanalyzer. Accurate quantification for sequencing applications was determined using a qPCR-based KAPA Biosystems Library Quantification kit (Kapa Biosystems). Each library was diluted to a final concentration of 10 nM and pooled equimolar prior to clustering. In all samples, 150 bp paired-end (PE150) sequencing was performed. The experimental technical process is shown in the Figure 1A. The technical process of analysis is shown in the Figure 1B.

### Differential mRNA, circRNA, and lncRNA Screen and Clustering Analysis

For analyzing of whole-transcriptome (mRNA, circRNA, and lncRNA) expression, the reads were first mapped to the latest UCSC transcript set using Bowtie2 (version 2.1.0) (Langmead and Salzberg, 2012), and the gene expression level was estimated using RSEM (version 1.2.15) (Li and Dewey, 2011). We used the transcripts set form Lncipedia^1^ for lncRNA expression analysis. Trimmed mean of *M*-values was used to normalize gene expression. Differentially expressed RNAs were identified using the edgeR program (Robinson et al., 2010). RNAs showing altered expression with *P* < 0.05 and fold change (FC) > 1.5 were considered differentially expressed. For circRNA expression analysis, the reads were genome mapped using STAR software (Dobin et al., 2013), and DCC software (Cheng et al., 2016) was used to identify the circRNA and to estimate the circRNA expression. Trimmed mean of *M*-values was used to normalize the expression. Differentially expressed RNAs were identified using the edgeR program, and miRanda (Enright et al., 2003) was used to predict the miRNA target of the circRNA. R was used to generate the figure.

### GO, KEGG and Reactome Enrichment Analysis

GO and KEGG analysis were used to determine key biological processes and signaling pathways during CCH process. Gene functions were classified into three subgroups: biological process (BP), cellular component (CC), and molecular function (MF). GO analysis^2^ was conducted to construct meaningful annotation of genes and gene products in a wide variety of organisms. GO terms with a *p* < 0.05 were selected and integrated using Venn analysis. The top 15 enriched GO terms among the groups were ranked by fold enrichment, and the enrichment scores were presented.

KEGG pathway/Reactome enrichment analysis was performed to generate pathway clusters on the molecular interaction and reaction networks in differentially regulated gene profiling that covered present knowledge. The −log10 (*P*-value) denotes the significance among differentially expressed RNAs.

### Ingenuity Pathway Analysis

Ingenuity pathway analysis was used to decipher mRNA expression patterns related functions, diseases and networks about VD induced by CCH. The mRNA expression data obtained from three samples of CCH 4W and CCH 8W tissues and four normal controls were imported into the IPA Tool (Ingenuity H Systems, Redwood City, CA, United States)^3^. The IPA tool computes a score for each network according to the fit of the set of supplied focus genes. These scores indicate the likelihood of focus genes belonging to a network versus those obtained by chance. The canonical pathways generated by IPA are the most significant for the uploaded dataset. Fischer’s exact test with FDR option was used to calculate the significance of the canonical pathway. Based on the Ingenuity Knowledge Base of different networks, BP and/or diseases were then algorithmically generated based on the connectivity of genes within the datasets. Comparisons were performed between the CCH groups and the sham group. The mRNAs showing significant differences in expression levels between groups will be submitted to IPA for human diseases and disorders under the molecular and cellular functions categories and pathway analysis.

### Co-expression Networks of mRNA–circRNA–miRNA and mRNA–lncRNA

The co-expression networks were constructed based on the correlation analysis between the differentially expressed mRNAs, circRNAs, and lncRNAs, and the expression of these RNAs was analyzed by Pearson’s correlation coefficient. The absolute coefficient value of 0.8 between RNAs was considered relevant for network construction. *P*-value < 0.05 was considered statistically significant.

### Quantitative Real-Time Polymerase Chain Reaction

Real-time polymerase chain reaction (qPCR) was performed to validate the results of the transcriptome analyses. cDNA (2 μl) was quantified in duplicate for each sample using a Light Cycler 480 SYBR Green I master kit (Roche) on a Light Cycler 480 II according to the manufacturer’s instruction. Cycling conditions were: 95°C for 10 min, 40 cycles of 95°C for 15 s and 60°C for 60 s. Melt curve cycles immediately followed, and the conditions were: 95°C for 5 s, 60°C for 1 min, and then a gradual temperature rise to 95°C at a rate of 0.3°C/s followed by 60°C for 15 s. Melt curve analysis was performed to verify primer specificity, and all primers were tested in a dilution series before use. For Cyr61 mRNA, *n* = 9 in each group, for other mRNAs, *n* = 6 in each group. Data are displayed as FC above proliferative condition mRNA levels using 2^∧(ΔΔ*Ct*)^ values. Primer sequences were described as follows:

Cyr61-S: CATCTCCACACGAGTTACCAA
Cyr61-A: CGGACTGGTTCTGGGGATTTC
NR4A2-S: CTGTCAGCATTACGGTGTTCG
NR4A2-A: CTTCGGTTTTGAGGGTAGACG
PDE4C-S: CTTGAAGACACCAACAAATGG
PDE4C-A: CCTGGAGAACGCTGAAGATAA
Apold1-S: TCTACTTCATCGTCTTCTTCGG
Apold1-A: CTCCAGGCTCTCAGACAGTTT
Atf3-S: ATTCGCCATCCAGAACAAGC
Atf3-A: AGCAGCAATTTTGTTTCTTTCC
Iift1-S: GCTACCACCTTCACAGCAACC
Iift1-A: ATCCTCACTTCCAAATCAGGTAT
Iift3-S: GAAGGATGGACACGCCTAAAG
Iift3-A: CCAAGGCATCTTCAACCAAC
R-actin-S: TGCTATGTTGCCCTAGACTTCG
R-actin-A: GTTGGCATAGAGGTCTTTACGG

### Western Blot Analysis

Proteins expression of fresh cortical tissue samples were analyzed by western blotting. All tissues were lysed in radioimmunoprecipitation assay buffer, and total protein concentrations were determined with a BCA Protein Assay Kit (Thermo Scientific). Total protein (10–20 μg) was loaded for each sample into pre-cast 4–12% bis-tris gels and run with 3-(*N*-morpholino) propanesulfonic acid buffer (Invitrogen). Gels were transferred onto polyvinylidene fluoride or polyvinylidene difluoride membranes (Millipore). Antigen-specific primary antibodies were incubated overnight at 4°C and detected with species-specific horseradish-peroxidase-conjugated secondary antibodies. An ECL Western Blotting Detection kit (GE Healthcare) was used to obtain a chemiluminescence signal, which was detected using Amersham Hyperfilm ECL (GE Healthcare). Bands of interest were normalized to β-actin (1:3000, Abcam) for a loading control. The following antibodies were used in this study: Caspase 1+P10+P12 (1:3000, Abcam); HIF1α (1:1000, Abcam); TNF α (1:1000, Abcam); RIPK1 (1:1000, Abcam); RIPK 3 (1:1000, Abcam); pRIPK3 (1:1000, Signalway Antibody); MLKL (1:1000, Signalway Antibody); Bcl-2 (1:1000, Abcam); Bax (1:2000, Abcam); Cleaved Caspase 3 (1:1000, Abcam); ZO-1 (1:1000, Merck-Millipore); Occludin (1:1000, Abcam); Cyr61 (1:500, Abcam). The protein bands were quantitatively analyzed with AlphaEase FC software.

### Statistical Analysis

Identical results for imaging/histopathology were obtained by the two readers (one, a major in medical imaging/pathology and the other, a major in neurology) (agreement); The final result was obtained after discussion between the two readers in disagreement (disagreement).

For brain tissue staining analysis/Morris water maze task/whole-transcriptome sequencing analysis, the cortical tissues of the three groups were numbered randomly. The experimenters and the analyzers did not know which groups the rats belonged to.

All non-bioinformatic data were analyzed using SPSS (Windows version 19.0, Abacus Concepts Inc., Chicago, IL, United States). The data were expressed as mean ± standard deviation (SD). All data are representative of at least three experiments performed in triplicate unless otherwise indicated. After assessing the normal distribution with the Shapiro–Wilk test, within-group data were analyzed with either one or two-way analysis of variance (ANOVA), Fisher’s least significant difference (LSD) test was used to compare the groups if the homogeneity of variance was determined, whereas Tamhane’s T2 test was used to compare the groups if the homogeneity of variance was not determined. Non-parametric Kruskal–Wallis *H* test was used for non-normal distributed variables followed by Nemenyi test was used for comparison between two groups. *P* < 0.05 was considered statistically significant.

## Results

### Cognitive Impairment

**FIGURE 2:**
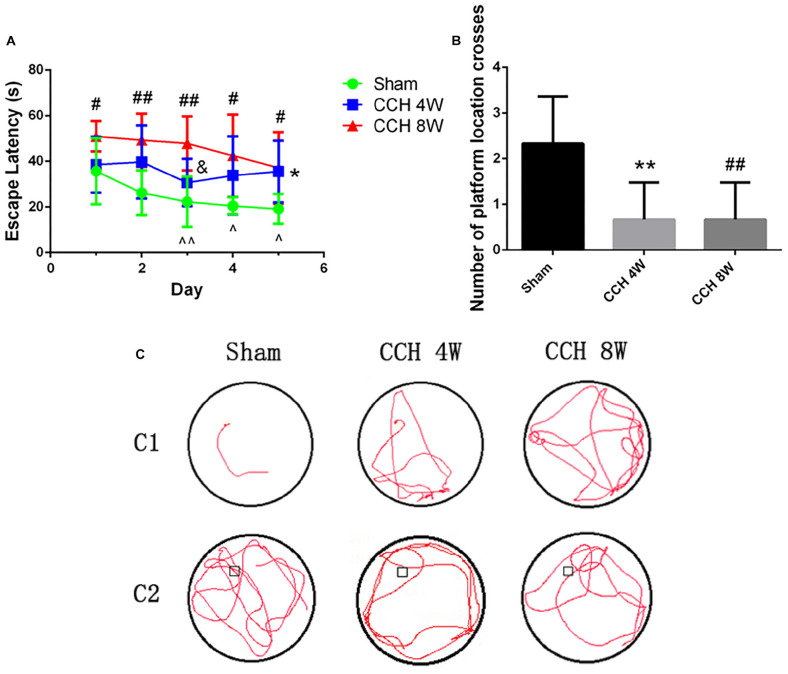
CCH exacerbates cognitive deficits. **(A)** The EL significantly differed between the CCH 4W or CCH 8W group and the sham-operated group. **(B)** The number of platform location crossings during a single 60 s probe trial. **(C)** Representative trajectories of EL **(C1)**, number of platform location crosses **(C2)**. *n* = 6 in each groups. Values are expressed as the mean ± SD. ^*^P < 0.05, ^∗∗^*P* < 0.01, CCH 4W compared with sham rats; ^#^*P* < 0.05, ^##^*P* < 0.01, CCH 8W compared with sham rats; ^&^*P* < 0.05, CCH 8W compared with CCH 4W rats; ^∧^*P* < 0.05, ^∧∧^*P* < 0.01, intergroup comparison of sham group.

The sham-operated rats quickly learned to find the hidden platform in the MWT (day 1 vs. day 3, *t* = 4.12, ^∧∧^*P* < 0.01; day 1 vs. day 4, *t* = 2.78, ^∧^*P* < 0.05; day 1 vs. day 5, *t* = 3.48, ^∧^*P* < 0.05) (Figure 2). The CCH 4W and CCH 8W groups took a longer time (escape latency [EL]) to find the hidden platform than did those in the sham (^*^P < 0.05, CCH 4W vs. the sham; #*P* < 0.05, ##*P* < 0.01, CCH 8W vs. the sham; &*P* < 0.05, CCH 4W vs. CCH 8W; Figure 2A). In the probe trial, the number of platform location crossings was lower in the CCH 4W and CCH 8W groups than in the sham group (^∗∗^*P* < 0.01, CCH 4W vs. the sham; ##*P* < 0.01, CCH 8W vs. the sham), indicating poor memory in the CCH rats (Figure 2B). Representative trajectories are shown in Figure 2C (Figure 2C1, the trajectory of EL; Figure 2C2, number of platform location crosses).

### Neuronal Morphology in the Cortex

**FIGURE 3:**
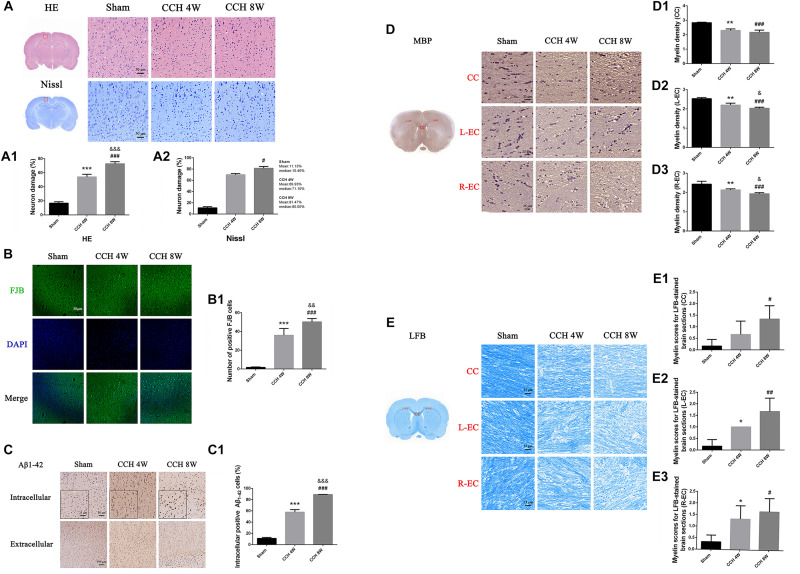
**(A)** HE and Nissl staining showed representative images of neuronal damage. Scale bar = 50 μm; magnification, 200×. **(A1)** Quantitative analysis results of HE staining. **(A2)** Quantitative analysis results of Nissl staining. **(B)** FJB staining showed representative images of neuronal degeneration. Scale bar = 50 μm; magnification, 200×. **(B1)** Quantitative analysis results of FJB staining. **(C)** Representative immunohistochemistry images of intracellular positive Aβ_1–42_ cells (magnification: 200×) and extracellular Aβ plaque burdens (magnification: 100×) in the cortex in the sham-operated, CCH 4W, and CCH 8W groups. **(C1)** Quantification of intracellular positive Aβ_1–42_ cells. **(D)** Diaminobenzidine staining of MBP in the CC, L-EC (left-EC), and R-EC (right-EC) areas. Scale bar = 25 μm. Magnification, 400×. **(D1–D3)** Myelin density of the CC, L-EC, R-EC. **(E)** Representative images of LFB-stained coronal sections (scale bar = 25 μm; magnification, 400×) and histological scoring. **(E1–E3)** Myelin scores of the CC, L-EC, R-EC. *n* = 3 in each groups. Values are expressed as the mean ± SD. ^*^P < 0.05, ^∗∗^*P* < 0.01, ^∗∗∗^*P* < 0.001, CCH 4W compared with sham rats; ^#^*P* < 0.05, ^##^*P* < 0.01, ^###^*P* < 0.001, CCH 8W compared with sham rats; ^&^*P* < 0.05, ^&&^*P* < 0.01, ^&⁣&&^*P* < 0.001, CCH 8W compared with CCH 4W rats.

HE and Nissl staining revealed significant morphological changes of neurons in the CCH groups (Figure 3A). We found more neuron damage happened in CCH groups, especially in CCH 8W (HE staining *F* = 266.46, *P* < 0.001; Sham vs. CCH 4W, ^∗∗∗^*P* < 0.001; Sham vs. CCH 8W, ^###^*P* < 0.001; CCH 4W vs. CCH 8W, &&&*P* < 0.001). The same as Nissl staining (*H* = 7.26, *P* < 0.05; Sham vs. CCH 8W, #*P* < 0.05). Quantitative analysis of morphological damage of neurons is shown in Figures 3A1,A2.

In sham-operated rats, almost no neurons were stained with FJB. However, FJB positive cells were easily identified in the CCH 4W and CCH 8W groups. Additionally, the CCH 8W group showed a greater number of denatured neurons than the CCH 4W group (*F* = 87.05, *P* < 0.001; Sham vs. CCH 4W, ^∗∗∗^*P* < 0.001; Sham vs. CCH 8W, ^###^*P* < 0.001; CCH 4W vs. CCH 8W, &&*P* < 0.01) (Figures 3B,B1).

### Intracellular Aβ-Positive Cells and Extracellular Aβ Plaques

Figure 3C shows representative images of intracellular Aβ-positive cells (200 magnification) and the extracellular Aβ plaque burden (100 magnification) in the sham, CCH 4W and CCH 8W groups. The number of intracellular Aβ1-42 positive cells was significantly higher in the CCH 4W and CCH 8W groups than in the sham (*F* = 608.86, *P* < 0.001; Sham vs. CCH 4w, ^∗∗∗^*P* < 0.001; Sham vs. CCH 8W, ^###^*P* < 0.001; CCH 4W vs. CCH 8W, &&&*P* < 0.001) (Figure 3C1). No significant differences in the extracellular Aβ1-42 plaque deposition were found among the three groups.

### Effects of CCH on Brain Myelin Status

After CCH, the loss of MBP was prominent in the CC (*F* = 32.94, *P* < 0.01; Sham vs. CCH 4W, ^∗∗^*P* < 0.01; Sham vs. CCH 8W, ^###^*P* < 0.001) and both ECs (L-EC: *F* = 35.00, *P* < 0.001; Sham vs. CCH 4W, ^∗∗^*P* < 0.01; Sham vs. CCH 8W, ^###^*P* < 0.001; CCH 4W vs. CCH 8W, &*P* < 0.05; R-EC: *F* = 19.00, *P* < 0.01; Sham vs. CCH 4W, ^∗∗^*P* < 0.01; Sham vs. CCH 8W, ^###^*P* < 0.001; CCH 4W vs. CCH 8W, &*P* < 0.05.) (Figures 3D,D1–D3).

CCH promoted changes in brain myelin status, including the increase in the demyelination score and the significant decline in LFB stain intensity (CC: Sham vs. CCH 8W, #*P* < 0.05; L-EC: *F* = 12.20, *P* < 0.01; Sham vs. CCH 4W, ^*^P < 0.05; Sham vs. CCH 8W, ##*P* < 0.01; R-EC: *F* = 5.78, *P* < 0.05; Sham vs. CCH 4W, ^*^P < 0.05; Sham vs. CCH 8W, #*P* < 0.05)(Figures 3E,E1–E3).

### White Matter Integrity in DTI

**FIGURE 4:**
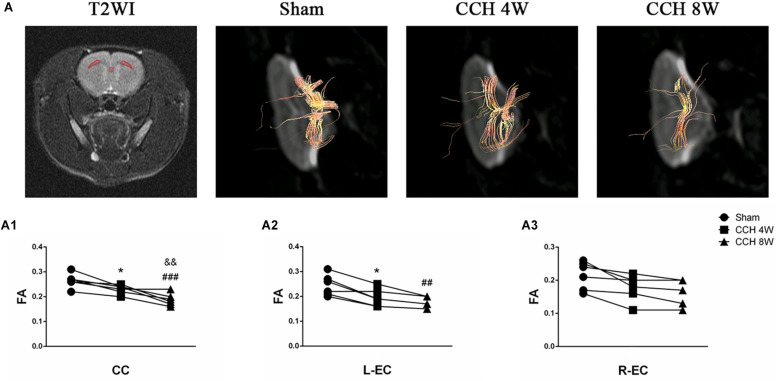
Evaluation of the white matter fibers by DTI. **(A)** Selected regions of interest in the CC and both ECs in T2WI. Representative tractography in three groups. **(A1–A3)** Quantitative analysis of FA value of DTI parameters. *n* = 6 in each groups. ^*^P < 0.05, CCH 4W compared with sham rats; ^##^*P* < 0.01, ^###^*P* < 0.001, CCH 8W compared with sham rats; ^&&^*P* < 0.01, CCH 8W compared with CCH 4W rats.

The areas with white matter tracts (CC and both ECs), were selected for T2WI (Figure 4A). Compared with the state of baseline, FA was significantly reduced in the CC (*F* = 15.01, *P* < 0.001; CCH 4W vs. baseline, ^*^P < 0.05; CCH 8W vs. baseline, ^###^*P* < 0.001; CCH 4W vs. CCH 8W, &&*P* < 0.01) and L-EC (*F* = 6.895, *P* < 0.01; CCH 4W vs. baseline, ^*^P < 0.05; CCH 8W vs. baseline, ##*P* < 0.01) in rats with BCCAO. Representative tractography (Figure 4A) and quantitative analyses (Figures 4A1–A3) revealed more abundant white matter tracts in the sham rats.

### Altered Protein Expression Associated With Neuron Death and BBB Damage After BCCAO

**FIGURE 5:**
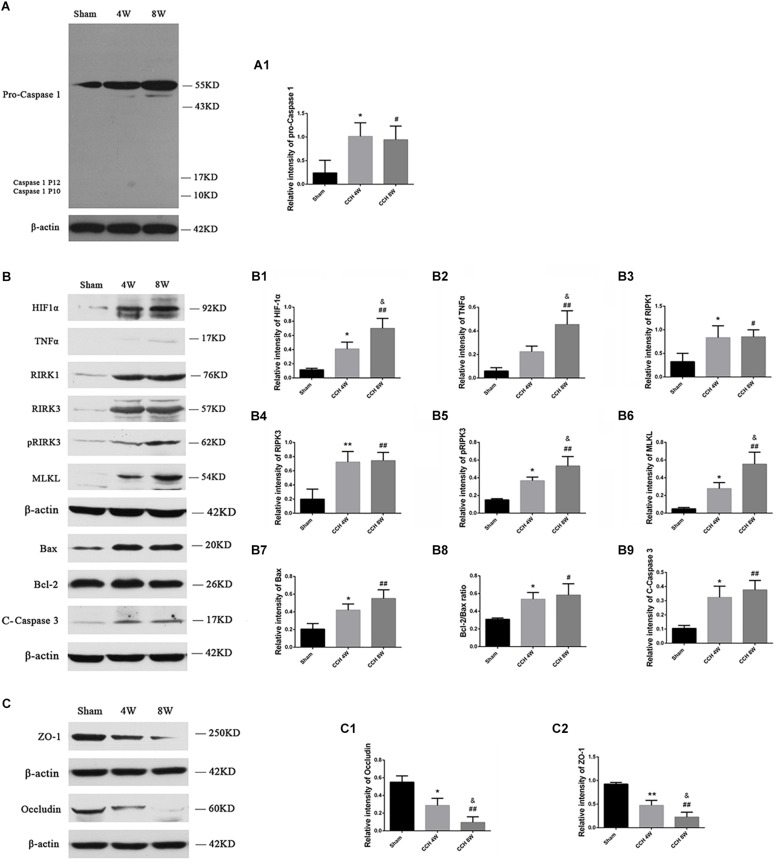
**(A)** Western blot analysis of pro-caspase 1, caspase 1 P12, and caspase 1 P10 protein in the cortex. **(B)** Western blot analysis of HIF-1α, TNFα, RIPK1, RIPK3, pRIPK3, MLKL, Bax, Bcl-2 and cleaved caspase 3 protein, and ratio of Bax/Bcl-2 in the cortex. **(C)** The expression of ZO-1 and occludin was lower in the CCH 4W and CCH 8W rats, especially in CCH 8W groups. Quantification of the band intensities is presented in the adjacent graphs **(A1,B1–B9,C1,C2)**. *n* = 3 in CCH 4W and CCH 8W rats, *n* = 2 in sham rats. Values are expressed as the mean ± SD. ^*^P < 0.05, ^∗∗^*P* < 0.01, CCH 4W compared with sham rats; ^#^*P* < 0.05, ^##^*P* < 0.01, CCH 8W compared with sham rats; ^&^*P* < 0.05, CCH 8W compared with CCH 4W rats.

HIF-1α protein expression was significantly higher in the CCH groups, especially in CCH 8W group than in the sham group (*F* = 17.53, *P* < 0.01; Sham vs. CCH 4W, ^*^P < 0.05; Sham vs. CCH 8W, ##*P* < 0.01; CCH 4W vs. CCH 8W, &*P* < 0.05) (Figure 5B), indicating the presence of CCH in the BCCAO rats.

Three types of cell death models, including pyroptosis, necroptosis, and apoptosis, were evaluated. Pyroptosis, identified by active caspase 1 P10 and P12 subunits, was not activated in CCH, even though the expression of pro-caspase 1 (Sham vs. CCH 4W, ^*^P < 0.05; Sham vs. CCH 8W, #*P* < 0.05) was significantly higher in the CCH groups relative to the sham group (Figure 5A). However, necroptosis, identified by active TNFα (*F* = 15.03, *P* < 0.01; CCH 8W vs. Sham, ##*P* < 0.01; CCH 8W vs. CCH 4W, &*P* < 0.05), RIPK1 (CCH 4W vs. Sham, ^*^P < 0.05; CCH 8W vs. Sham, #*P* < 0.05), RIPK3 (*F* = 11.64, *P* < 0.05; CCH 4W vs. Sham, ^∗∗^*P* < 0.01; CCH 8W vs. Sham, ##*P* < 0.01), pRIPK3 (*F* = 16.65, *P* < 0.01; CCH 4W vs. Sham, ^*^P < 0.05; CCH 8W vs. Sham, ##*P* < 0.01; CCH 8W vs. CCH 4W, &*P* < 0.05), and MLKL (*F* = 16.91, *P* < 0.01; CCH 4W vs. Sham, ^*^P < 0.05; CCH 8W vs. Sham, ##*P* < 0.01; CCH 8W vs. CCH 4W, &*P* < 0.05) (Figure 5B), and apoptosis, identified by apoptosis markers including Bax (*F* = 10.56, *P* < 0.05; CCH 4W vs. Sham, ^*^P < 0.05; CCH 8W vs. Sham, ##*P* < 0.01), Bax/Bcl-2 ratio (*F* = 5.53, *P* < 0.05; CCH 4W vs. Sham, ^*^P < 0.05; CCH 8W vs. Sham, #*P* < 0.05) and cleaved caspase 3 (*F* = 10.63, *P* < 0.05; CCH 4W vs. Sham, ^*^P < 0.05; CCH 8W vs. Sham, ##*P* < 0.01) (Figure 5B), increased 4th and 8th weeks after BCCAO. In addition, the expression of occludin (*F* = 18.75, *P* < 0.01; CCH 4W vs. Sham, ^*^P < 0.05; CCH 8W vs. Sham, ##*P* < 0.01; CCH 8W vs. CCH 4W, &*P* < 0.05) and ZO-1 (*F* = 28.39, *P* < 0.01; CCH 4W vs. Sham, ^∗∗^*P* < 0.01; CCH 8W vs. Sham, ##*P* < 0.01; CCH 8W vs. CCH 4W, &*P* < 0.05) decreased in the CCH 4W and CCH 8W groups (Figure 5C). Quantification of the band intensities is presented in the adjacent graphs (Figures 5A1,B1–B9,C1,C2).

### Differentially Expressed lncRNA, mRNA, and circRNA Profiles

**FIGURE 6:**
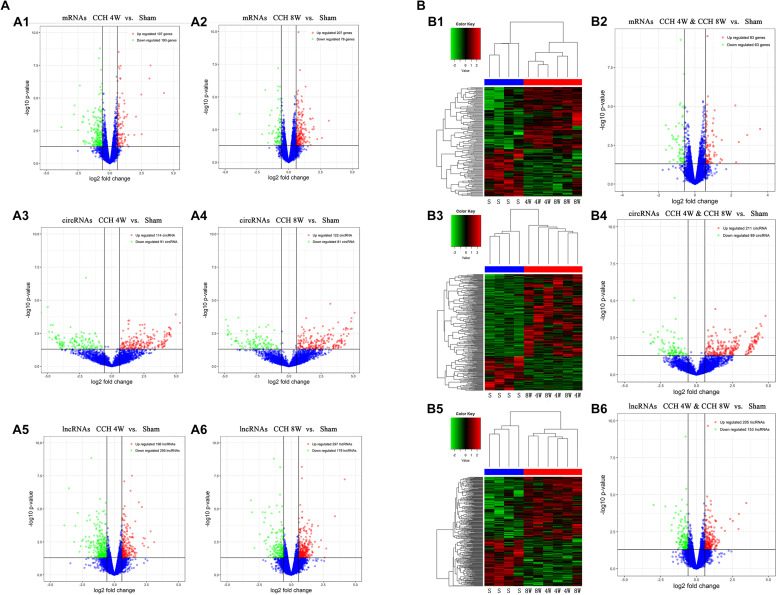
Assessment of circRNA, lncRNA, and mRNA expression profiles in the screening phase. **(A) (A1**,**A2)** Expression profiles (volcano plots) of mRNAs in the CCH 4W and CCH 8W rats, compared with the sham rats (**A1**, CCH 4W; **A2**, CCH 8W). **(A3**,**A4)** Expression profiles (volcano plots) of circRNAs in the CCH 4W and CCH 8W rats, compared with the sham rats (**A3**, CCH 4W; **A4**, CCH 8W). **(A5**,**A6)** Expression profiles (volcano plots) of lncRNAs in the CCH 4W and CCH 8W rats, compared with the sham rats (**A5**, CCH 4W; **A6**, CCH 8W). **(B) (B1**,**B2)** Expression profiles of mRNAs in CCH 4W&CCH 8W rats compared with the sham rats (**B1** for clustering analysis and **B2** for volcano plots). **(B3**,**B4)** Expression profiles of circRNAs in CCH 4W&CCH 8W rats compared with the sham rats (**B3** for clustering analysis and **B4** for volcano plots). **(B5**,**B6)** Expression profiles of lncRNAs in CCH 4W&CCH 8W rats compared with the sham rats (**B5** for clustering analysis and **B6** for volcano plots). S, sham; 4W, CCH 4W; 8W, CCH 8W; genes, mRNAs.

A total of 11806 mRNAs were detected, and 300 mRNAs were differentially expressed in three CCH 4W tissues compared with four controls (FC ≥ 1.5, *P* < 0.05, and FDR < 0.05). Among the 300 mRNAs, 107 were upregulated, and 193 were downregulated (Figure 6A1). The microarray analysis identified a total of 4109 circRNAs and 14472 lncRNAs, which were dysregulated in CCH. Filtering analysis identified 205 differentially expressed circRNAs between CCH 4W and control tissues; among these circRNAs, 114 were upregulated, and 91 were downregulated (Figure 6A3). In addition, 491 lncRNAs were shown to be differentially expressed: 196 lncRNAs were upregulated, and 295 lncRNAs were downregulated (FC ≥ 1.5, *P* < 0.05, and FDR < 0.05; Figure 6A5). Coding gene profiles revealed that 3 mRNAs had FC ≥ 10, Amy1a and LOC100134871 were the most upregulated (FC = 19) and downregulated mRNA (FC = −452), respectively. In total, 19 circRNAs, including 1 upregulated circRNA and 18 downregulated circRNAs, had FC ≥ 10. CircKdm4c (FC = 31) and CircMyo19 (FC = −59) were the most upregulated and downregulated circRNAs, respectively. In addition, five lncRNAs displayed FC ≥ 10. LncOlr1718 (FC = 660) was the most upregulated lncRNA, and lncStyxl1 (FC = −122) was the most downregulated lncRNA.

In total, 283 differentially expressed mRNAs were identified in three CCH 8W tissues compared with four controls (FC ≥ 1.5, *P* < 0.05, and FDR < 0.05). Among the 283 mRNAs, 207 were upregulated, and 76 were downregulated (Figure 6A2). In addition, 203 differentially expressed circRNAs were identified between CCH 8W and control tissues; among the 203 circRNAs, 122 were upregulated, and 81 were downregulated (Figure 6A4). Moreover, 476 differentially expressed lncRNAs were found; among them, 297 and 179 lncRNAs were upregulated and downregulated, respectively (FC ≥ 1.5, *P* < 0.05, and FDR < 0.05; Figure 6A6). The coding gene profile revealed that only 1 mRNA, RT1-CE11, had FC ≥ 10, and RT1-CE11 was the most downregulated mRNA (FC = −14). Furthermore, 11 circRNAs, including 5 upregulated circRNA and 6 downregulated circRNAs, displayed FC ≥ 10. circRbl2 (FC = 29) and CircMyo19 (FC = −59) were the most upregulated and downregulated circRNAs, respectively. Two lncRNAs, including 1 upregulated and 1 downregulated lncRNAs, displayed FC ≥ 10. LncOlr1735 (FC = 17) was the most upregulated lncRNA, and lncCkmt2 (FC = −97) was the most downregulated lncRNA.

In total, 146 differentially expressed mRNAs were identified in six CCH samples compared with four controls (FC ≥ 1.5, *P* < 0.05, and FDR < 0.05). Among the 146 mRNAs, 83 and 63 mRNAs were upregulated and downregulated, respectively (Figures 6B1,B2). Filtering analysis identified 300 differentially expressed circRNAs in the CCH samples compared with controls; among them, 211 were upregulated, and 89 were downregulated (Figures 6B3,B4). In addition, 358 differentially expressed lncRNAs were identified; among these lncRNAs, 205 and 153 lncRNAs were upregulated and downregulated, respectively (FC ≥ 1.5, *P* < 0.05, and FDR < 0.05; Figures 6B5,B6). The coding gene profile revealed that 1 mRNA, identified as Amy1a, had FC ≥ 10, and Amy1a was the most upregulated mRNA (FC = 11). Moreover, 49 circRNAs, including 46 upregulated lncRNA and 3 downregulated circRNAs, displayed FC ≥ 10. CircNpepps (FC = 27) and CircMyo19 (FC = −59) were the most upregulated and downregulated circRNAs, respectively. Three upregulated lncRNAs displayed FC ≥ 10, and LncOlr1718 (FC = 332) was the most upregulated lncRNA.

### GO, KEGG, and Reactome Enrichment Analysis in the CCH Groups

#### GO Enrichment Analysis

**FIGURE 7:**
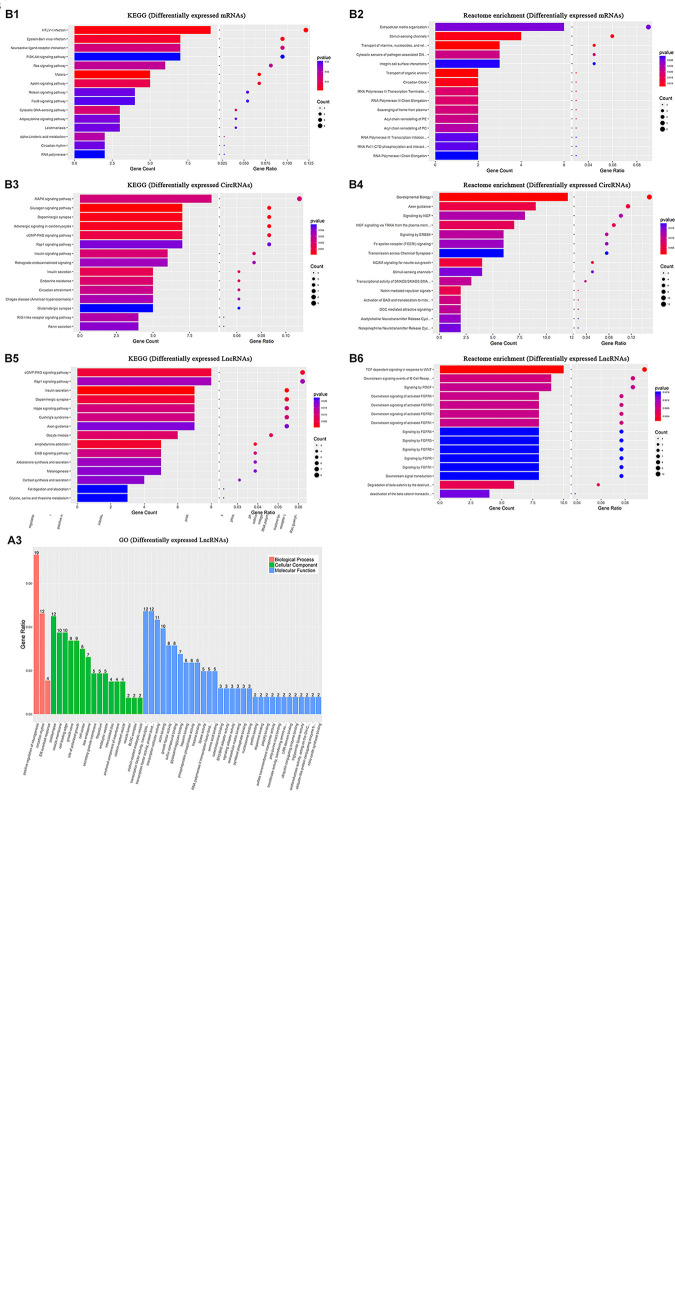
GO, KEGG, and Reactome enrichment analysis. In six CCH samples (CCH 4W and CCH 8W) compared with four sham controls: **(A)** GO annotation of upregulated and downregulated mRNAs **(A1)**, circRNAs **(A2)**, and lncRNAs **(A3)** of BPs, CCs, MFs. **(B)** KEGG analysis of upregulated and downregulated mRNAs **(B1)**, circRNAs **(B3)**, and lncRNAs **(B5)** with top 15 enrichment scores. **(B)** Reactome enrichment analysis of upregulated and downregulated mRNAs **(B2)**, circRNAs **(B4)**, and lncRNAs **(B6)** with top 15 enrichment scores.

The changes in mRNA levels were associated with BP in response to peptides in six CCH samples (CCH 4W and CCH 8W) compared with four controls: CC was associated with extracellular matrix and the apical part of the cell, and MF was associated with carboxylic acid binding (Figure 7A1). The apparent changes in circRNA associated with BP were single-organism behavior. CC was associated with postsynaptic elements, and MF was associated with protein serine/threonine kinase activity (Figure 7A2). The apparent changes in lncRNA were associated with the positive regulation of neurogenesis. CC was associated with postsynaptic elements, and MF was associated with transcription factor activity, transcription factor binding, and protein binding (Figure 7A3).

#### KEGG Enrichment Analysis

##### Differentially expressed mRNAs

Our data showed that the top three pathways were HTLV-1 infection, Epstein-Barr virus infection, and neuroactive ligand-receptor interaction in the six CCH samples compared with four controls (Figure 7B1).

##### Differentially expressed circRNAs

The MAPK signaling pathway, glucagon signaling pathway, and dopaminergic synapse were the top three pathways (Figure 7B3).

##### Differentially expressed lncRNAs

The cGMP-PKG signaling pathway, Rap1 signaling pathway, and the insulin secretion pathway were the top three pathways (Figure 7B5).

#### Reactome Enrichment Analysis

##### Differentially expressed mRNAs

The top 5 pathways identified were extracellular matrix organization, stimuli-sensing channels, transport of vitamins, nucleosides, and related molecules, including cytosolic sensors of pathogen-associated DNA, in the six CCH samples compared with four controls, and integrin cell surface interactions also played a crucial role in the pathogenesis of CCH (Figure 7B2).

##### Differentially expressed circRNAs

Developmental biology, axon guidance, signaling by NGF, NGF signaling via TRKA from the plasma membrane, and signaling by ERBB4 were the top 5 pathways in circRNAs (Figure 7B4).

##### Differentially expressed lncRNAs

TCF dependent signaling in response to WNT, downstream signaling events of B cell receptor, signaling by PDGF, downstream signaling of activated FGFR3 and FGFR4 were the top 5 pathways in lncRNAs (Figure 7B6).

### Construction of the circRNA–miRNA and mRNA–circRNA–miRNA Co-expression Network in the CCH Groups

**FIGURE 8:**
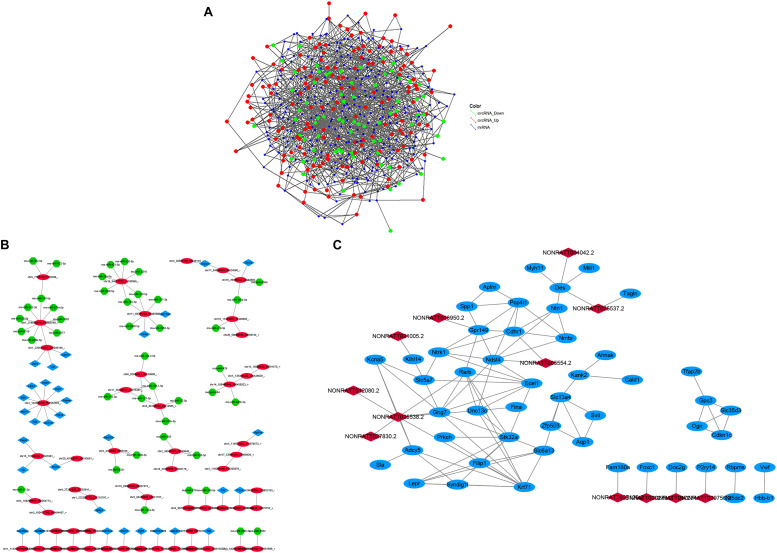
**(A)** The circRNA-miRNA co-expression network. circRNA and miRNA were indicated with three colored deformed diamonds. The red color represents the high expression, green color represents the low expression of circRNA, blue color represents miRNA. The size of diamonds represents FC of circRNAs, and a larger size represents a higher FC. **(B)** The mRNA–circRNA–miRNA co-expression network. Blue square nodes represent mRNAs, red circle nodes represent circRNAs, green shape nodes represent miRNAs. Solid lines represent relationships. **(C)** The lncRNA-mRNA co-expression network. Blue circle nodes represent mRNAs, red square nodes represent lncRNAs. Solid lines represent relationships.

A circRNA–miRNA co-expression network was constructed based on the microarray analysis in the six CCH samples compared with four controls. A network map containing 283 circRNAs, 749 miRNAs, and 7223 relationships was constructed (Figure 8A). A mRNA–circRNA–miRNA co-expression network map containing 32 mRNAs, 55 circRNAs, and 42 miRNAs was constructed, and 34 relationships between circRNA and mRNA and 50 relationships between circRNA and miRNA were observed (Figure 8B).

### Co-expression of lncRNAs–mRNAs and Function Prediction

The co-expression network suggests that one lncRNA may correlate with multiple mRNAs in CCH samples compared with the sham. We chose some significantly expressed coding genes and lncRNAs in the CCH groups to build a network, according to the degree of correlation (Figure 8C).

As we known, mRNAs are implicated in a number of BPs. For example, the network revealed that downregulated lncRNAs, identified as NONRATT016029.2 and NONRATT016027.2, were positively correlated with the Ckmt2. The network may suggest that the regulation between lncRNAs and mRNAs, which is implicated in CCH.

### IPA in the CCH Groups

#### Biological Functions Associated With CCH

The most significant disorders associated with CCH-correlated mRNAs were related to cancer, organismal injury and abnormalities, infectious disease, hematological disease, immunological disease, metabolic disease, and neurological disease (Table 1, upper panel), and 15 focus genes were related to neurological disease. Cell death and survival were among the most significant molecular and cellular function categories (Table 1, bottom panel).

**TABLE 1:** Biological functions associated with chronic cerebral hypoperfusion.

**Network**	**Top functions**	***p*-value**	**Focus mRNAs**
**Diseases and disorders**			
1	Cancer, organismal injury, and abnormalities	<0.001	129
2	Infectious diseases	<0.001	27
3	Hematological disease, immunological disease	<0.01	27
4	Metabolic disease	<0.01	17
5	Neurological disease	<0.001	15
**Molecular and cellular functions**			
1	Cell death and survival	<0.001	38
2	Cellular movement	<0.001	28
3	Gene expression	<0.01	24
4	Cellular growth and proliferation	<0.001	16
5	Cell-to-cell signaling and interaction	<0.01	16

*The most significant disease and disordered biological functions associated with CCH-correlated mRNAs were related to cancer (upper panel). Cell death and survival were the top significant molecular and cellular functional categories (bottom panel).*

### Canonical Pathways Analysis

To gain further insights into the pathogenesis of CCH, we analyzed CCH-correlated genes to elucidate dominant canonical pathways. The top 10 canonical signaling pathways were categorized (Table 2).

**TABLE 2:** Top 10 significantly changed canonical signaling pathways in CCH rats compared with sham controls.

**Ingenuity canonical pathways**		**−log (*p*-value)**
1	Glycine betaine degradation	2.71
2	Role of macrophages, fibroblasts and endothelial cells in rheumatoid arthritis	2.35
3	Mitochondrial L-carnitine shuttle pathway	2.24
4	Acute phase response signaling	2.24
5	p38 MAPK signaling	2.06
6	Atherosclerosis signaling	2.01
7	Iron homeostasis signaling pathway	1.97
8	Adipogenesis pathway	1.92
9	Caveolar-mediated endocytosis signaling	1.92
10	EIF2 signaling	1.84

*The top 10 canonical signaling pathways were categorized in order based on the level of statistical significance.*

### Network Analysis

**FIGURE 9:**
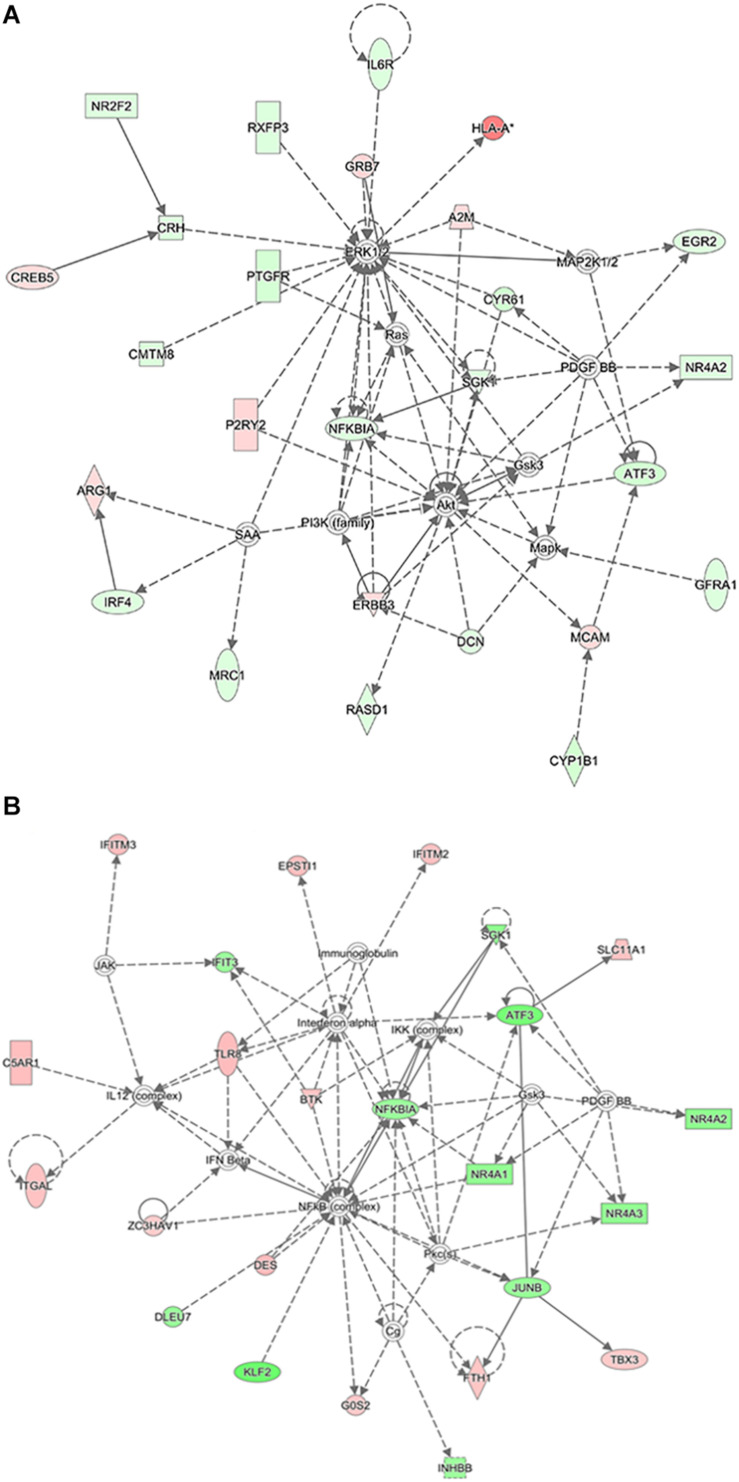
Ingenuity pathway analysis. IPA showed top interaction networks for differentially regulated mRNAs in CCH 4W **(A)** and CCH 8W **(B)** rats. Those highlighted with red color are upregulated mRNAs and those with green are downregulated mRNAs.

Figure 9 shows the molecular networks of differentially expressed genes analyzed by the IPA. These genes were involved in the ERK1/2, AKT, PI3K, NF-κBIA, ras, and SGK1 signaling pathways in the CCH 4W group (Figure 9A) and the NF-κB (complex), NF-κBIA, interferon-α, and interferon-β signaling pathways in the CCH 8W group (Figure 9B).

### Function of Cysteine Rich Angiogenic Inducer 61 (Cyr61)

**FIGURE 10:**
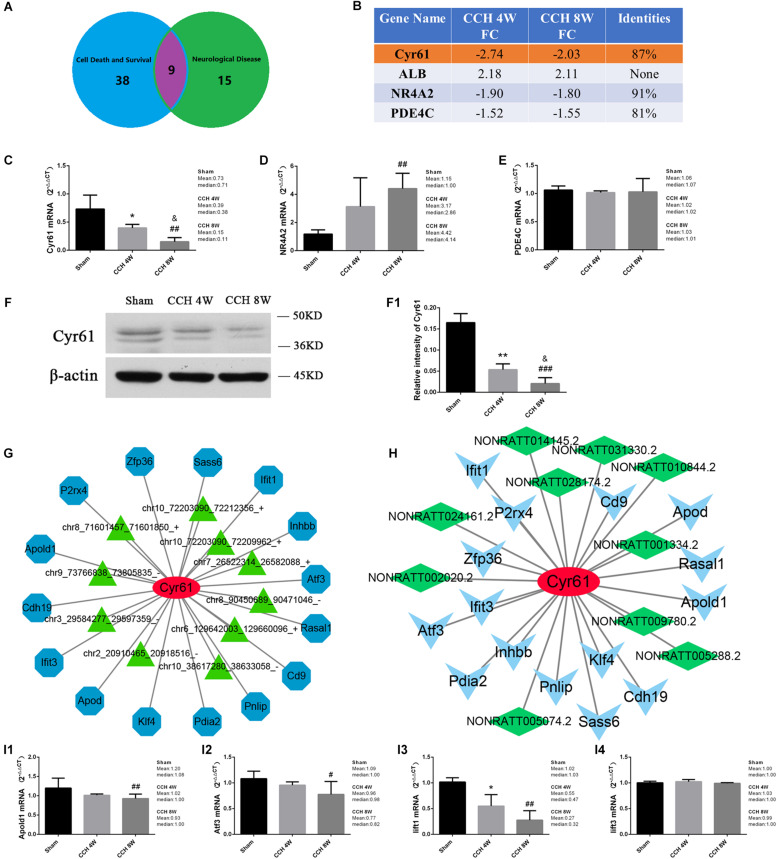
**(A)** The common mRNAs in BF in neurological diseases and cell survival and death, CCH. 4W&CCH 8W compared with sham rats. **(B)** Conservative analysis. **(C–E)** qPCR analysis of expression of Cyr61, NR4A2, PDE4C mRNA. **(F)** Western blot analysis of Cyr61 protein. **(F1)** Quantitative analysis of western blot. **(G)** Co-expression network of Cyr61 mRNA with the associated mRNAs and circRNAs. Blue nodes represent mRNAs, and green triangle nodes represent circRNAs. Solid lines represent relationships. **(H)** Co-expression network of Cyr61 mRNA with the associated mRNAs and lncRNAs. Blue triangles represent mRNAs, and green squares represent lncRNAs. Solid lines represent relationships. **(I1–I4)** qPCR analysis of Apold1, Atf3, Iift1, Iift3 mRNA. Values are expressed as the mean ± SD. ^*^P < 0.05, ^∗∗^*P* < 0.01, CCH 4W compared with sham rats; ^#^*P* < 0.05, ^##^*P* < 0.01, ^###^*P* < 0.001, CCH 8W compared with sham rats; ^&^*P* < 0.05, CCH 8W compared with CCH 4W rats; ns, not significant.

Nine mRNAs were involved in neurological disease and cell death and survival (Figure 10A). Four mRNAs had the same trend in the CCH 4W and CCH 8W groups: Cyr61, ALB, NR4A2, and PDE4C. After comparing the 4 mRNAs in rats with those in humans by Blast^®^^4^, Cyr61 may be associated with the neuronal death caused by CCH in the cerebral cortex (FDR > 2; Identified score, 87%; Figure 10B). The expression of mRNAs (Cyr61, NR4A2, PDE4C) were verified by qPCR, only the Cyr61 mRNA expression trend was consistent with the sequencing results (*H* = 22.37, *P* < 0.001; CCH 4W vs. Sham, ^*^P < 0.05; CCH 8W vs. Sham, ##*P* < 0.01; CCH 8W vs. CCH 4W, &*P* < 0.05) (Figures 10C–E). Western blotting analysis demonstrated that the expression of Cyr61 showed an opposite trend with increased time of CCH (*F* = 53.96, *P* < 0.001; CCH 4W vs. Sham, ^∗∗^*P* < 0.01; CCH 8W vs. Sham, ^###^*P* < 0.001; CCH 8W vs. CCH 4W, &*P* < 0.05) (Figures 10F,F1). Cyr61 was associated with 15 mRNAs, 10 circRNAs, and 10 lncRNAs (Figures 10G,H). Four mRNAs (Apold1, Atf3, Iift1, Iift3) of 15 mRNAs were verified by qPCR, and there were synergy effect between Apold1 (*H* = 9.59, *P* < 0.01; CCH 8W vs. Sham, ##*P* < 0.01), Atf3 (*H* = 6.38, *P* < 0.05; CCH 8W vs. Sham, #*P* < 0.05), Iift1 (*H* = 11.70, *P* < 0.01; CCH 4W vs. Sham, ^*^P < 0.05; CCH 8W vs. Sham, ##*P* < 0.01) and Cyr61 in CCH (Figures 10I1–I4).

## Discussion

Vascular dementia is one of the most common causes of dementia after Alzheimer’s disease (AD), which occurs in about 15∼20% of all the dementia. VD may occur post-stroke, due to idiopathic or genetic small vessel disease or because of chronic hypoperfusion due to carotid artery stenosis/occlusion. However, no licensed diagnosis and treatments are available (Chmayssani et al., 2007; Iadecola, 2013; O’Brien and Thomas, 2015; Choi et al., 2016). This is mainly because clinically VD has diversity and complexity in cerebrovascular pathologic conditions, risk factors, progression, and severity of disease (Du et al., 2017). CCH is a common consequence of diversiform cerebral vascular disorders and hemodynamic changes. Increasing evidence has suggested that CCH state/chronic cerebral ischemia is a premonitory symptom, which is associated with occurrence of VD (Du et al., 2017; Duncombe et al., 2017). A clinical population-based study indicated that a lower baseline cerebral hypoperfusion was associated with accelerated cognitive decline and an increased risk of dementia (Wolters et al., 2017). Accordingly, it is essential to get a better understanding of CCH induced VD (Du et al., 2017). The limitations associated with current clinical diagnosis and treatment are related to limitations in knowledge of the molecular mechanisms of CCH involved. Considering the difficulty and ethical nature of the human brain tissue’s provenance, laboratory rats have been used for decades to model diseases of the human central nervous system (CNS). BCCAO operation is currently recognized as a appropriate model of CCH to investigate the pathogenesis and therapeutics, especially to examine cognitive decline associated with cerebrovascular diseases (Du et al., 2017; Zhang T. et al., 2017; Zou et al., 2018; Kato et al., 2019; Mao et al., 2019). The chronic hypoperfusive conditions related to vascular cognitive impairment show a ∼20–30% decrease in blood flow, the level of hypoperfusion induced by BCCAO rat model was consistent with this trend (Duncombe et al., 2017). And previous studies considered that 4–8 weeks after BCCAO operation was the later stage (chronic phase) of the CCH process (Ma et al., 2015; Zhang X. et al., 2017; Kato et al., 2019). Therefore, the CCH model was available in this study.

In this research, from a brand-new perspective, WMD, different ways of cortical neuron death and whole-transcriptome analysis of rats with CCH were evaluated comprehensively.

### Disintegration of the White Matter

Cerebral white matter is comprised of nerve fibers interconnecting neurons in the cerebral cortex or the deep structures (Tomimoto, 2015). Emerging evidence indicates that reduced cerebral perfusion may contribute to several diseases characterized by WMD. WMD is another reflection of CCH and cerebral hypoxia, and a relationship is found between cognitive impairment and WMD (Iadecola, 2013; Choi et al., 2016). In addition, the imaging changes of hypoperfusion dementia including white matter lesions (O’Brien and Thomas, 2015).

DTI is highly sensitive to the directional diffusivities of water where tissues are oriented according to particular directions of white matter tracts (Choi et al., 2016). In our study, considering that white matter tracts are the most abundant in the CC and both ECs in the rat brain, reduced FA in the CC and both ECs may suggest the presence of concomitant axonal damage in the brain after CCH. Additionally, MBP is located in the dense myelin sheath and nucleus pulposus, LFB specifically binds to myelin; therefore, MBP and LFB staining can be used to assess the shape and monitor the change of the myelin sheath. The results indicate that WMD induced by CCH is characterized by disintegration of diverse white matter components. Considering the integrity of white matter, other mechanisms might be also involved in the disintegration of the white matter, such as neuron death. Different degrees of WMD may lead to morphological changes in cerebral cortex and its associated regions, the networks between neurons are impaired and their viability would be impaired.

### Cognitive Impairments, Aβ Deposition, BBB Injury and Neuron Death in CCH

Previous studies indicate that CCH is generally associated with a chain of disruption of homeostatic interactions, led to neuronal damage; experimental studies suggest that the damaging effects of such imbalances contribute to and exacerbate the course of VD (Jing et al., 2015; Du et al., 2017). The Morris water maze task examines the animals’ abilities to spatial learning and reference memory, making it ideal for many experimental models (Vorhees and Williams, 2006). Our study revealed cognitive impairments are induced by CCH, especially in CCH 8W. Spatial reference memory is thought to be related to not only the integrity of the hippocampus, but also cortex (Block and Schwarz, 1997). It had been reported that reference memory dysfunction will not occur until more than 60% of the neurons in the hippocampal CA1 sector are lost, and previous study showed that hippocampal neurons displayed significant damage since 8 weeks after BCCAO (Zou et al., 2018). CCH 4W might be in the transition progression of cognitive impairment. Therefore, change of biochemical indicators can also occur before severe cognitive impairment (Jing et al., 2015; Zou et al., 2018).

The number of intracellular Aβ-positive cells was greater in the CCH than the sham-operated, but Aβ plaque deposition was similar in these groups. Aβ_1–42_ was reported to enhance BBB permeability through reduction of tight junction proteins expression (Chan et al., 2018). In this study, the BBB was significantly damaged after CCH with decreased tight junction proteins including ZO-1 and Occludin. Aβ toxicity increases the formation of reactive oxygen species (ROS) and intracellular calcium levels, resulting in neuron death (Song et al., 2018). Considering Aβ plaque deposition is the remarkable pathological feature of AD, the pathogenesis and neuropathological changes caused by VD and AD are still different. In our study, we found intracellular Aβ deposition in CCH groups, which may be related to neuron death. A previous study indicated that extracellular Aβ plaque deposition occurred later than intracellular Aβ accumulation in the cortex of CCH rats (Zou et al., 2018). Therefore, extracellular Aβ deposition may occur after neurons’ death in later stage of CCH.

Additionally, significant neurodegeneration and neuron death were observed in the cortex. Apoptosis and necroptosis were both expressed in the CCH groups. Classically, cell death has been classified into apoptosis and necrosis. Apoptosis is a programmed form of cell death, but in the process of programmed cell death, the process of cell death can also be driven by other molecular pathways, which include programmed cell necrosis, such as necroptosis triggered by treatment with TNF (Linkermann and Green, 2014), inflammation related pyroptosis, or caspase 1-dependent cell death (Bergsbaken et al., 2009). The process of active pyroptosis was not detected in the CCH rats in the study. Apoptosis and necroptosis are not mutually exclusive, and both may occur in the same organ (Linkermann and Green, 2014). Salvatore et al. found that activated necroptosis induced hippocampus neuronal loss in AD (Caccamo et al., 2017). Even though hippocampus was demonstrated to have important role in cognitive function, from another perspective, CCH is induced by a moderate and continuous decrease in the CBF of cortex (Du et al., 2017), which leads to hypoxia of brain tissue followed by a series of neuropathological changes, which eventually lead to the impairment of cognitive function (Raz et al., 2016; Duncombe et al., 2017). Due to the cross-linking of the cortex and the hippocampus (Tomimoto, 2015), thus, CCH affects the hippocampus to a certain extent after the damage to the cortical-hippocampal communication network. Therefore, relationship between neuron death and transcriptional changes in cortical tissue have certain research value in the cognitive impairment induced by CCH. Additionally, WMD and neuron death may be the cause and effect of each other. Therefore, understanding the basic molecular mechanisms underlying the process of cortical neuron death in VD induced by CCH will be invaluable to the development of potential therapeutic approaches.

### Different Expression Profiles and Networks of the Whole Transcriptome in Chronic Cerebral Hypoperfusion

In this study, comparing CCH 4W group with CCH 8W group, some of the factors showed a statistically significant difference, but there were also a few factors showing non-significant changes, measured in brain tissues. The differences observed between macroscopic pathological phenomena and microscopic biochemical changes, highlight the importance of exploring the mechanism at a microscopic level.

Recently, progress has been made in understanding the formation and biogenesis of circRNAs, adding more evidence and possibilities to their biological applications. Studies have investigated the potential role of circRNAs as biomarkers of AD and tumors (Dou et al., 2016). In our study, the 2 most downregulated circRNAs were chr10_72203090_72212356_+ and chr10_72203090_72209962_+. Their target gene, Myo19, is involved in mitochondrial motility.

MicroRNAs (miRNA) are small (∼22 nt) single-stranded RNAs that are important regulators of several BPs, circRNAs regulate gene expression via specific miRNA binding sites (Vijayan et al., 2017; Wu et al., 2018). The circRNA–miRNA network may serve as a powerful regulation pathway in CCH. In our study, miRNAs were highly expressed in the CCH rats. In addition, miR-26b-targeted circRNAs were chr6_122804706_122817427_- and chr7_53979119_54012529_+; miR-195-targeted circRNAs were chr14_28418125_28536253_−, chr15_70769051_70832121_+, and chr20_14319169_14340940_−; miR-501-3p-targeted circRNAs were chr11_46896817_46915497_−, chr7_33866231_33886951_−, chr15_104270169_104283452_−, chr17_75579711_75616081_+, chr17_75588945_75604406_+ and chr4_8223839_8242710_−. The results suggest that CCH-related processes, such as mitochondrial motility, microglial activation and neurotoxicity, neuron degeneration and death, memory deficits, and white matter lesions, may be related to the circRNA–miRNA networks. It has already been proven that some miRNAs are functional in vascular cognitive impairment. For example, miRNA-195 prevents dendritic degeneration and neuron death in CCH rats (Chen et al., 2017); the TNFα–miR-501-3p–ZO-1 axis plays an important role in the pathogenesis of CCH induced memory deficits and WMD (Toyama et al., 2018).

lncRNA has been shown to regulate gene expression at various levels and has emerged as a major source of bio-targets for cancer therapeutics (Wu et al., 2018). The biological function of lncRNAs is multifaceted. The regulatory role of lncRNAs is not solitary but through a large complex network that involves mRNAs, miRNAs, and proteins. Several lncRNAs have been shown to be critical in regulating cellular processes and diseases, but many other functions of lncRNAs have not been elucidated (Dou et al., 2016). Therefore, the functional forecast of lncRNAs is based on the annotations of the co-expressed mRNAs’ function. The lncRNA–mRNA network showed that the downregulated lncRNAs, NONRATT016029.2 and NONRATT016027.2, were positively correlated with the Ckmt2 gene. The creatine kinase phosphagen system, including Ckmt2, is fundamental to cellular energy homeostasis, and decreased creatine kinase activity is a characteristic of ischemia-reperfusion injury and cell death (Zervou et al., 2017).

### Biological Function Analysis

Advanced bioinformatic tools including GO analysis and KEGG pathway analysis, have been applied to determine key biological processes and signaling pathways during diseases (Yu et al., 2016).

The KEGG pathway analysis of differentially expressed mRNAs revealed neuroactive ligand-receptor interaction, PI3K-Akt signaling and ras signaling pathways could play pivotal roles in the pathogenesis of CCH. The most significant GO items were response to peptide, extracellular matrix and carboxylic acid binding. Reactome enrichment analysis found that the top pathways were extracellular matrix organization, cytosolic sensors of pathogen-associated DNA, integrin cell surface interactions, et al.

The KEGG pathway analysis of differentially expressed circRNAs and lncRNAs revealed that MAPK signaling, glucagon signaling, dopaminergic synapse cGMP-PKG signaling could play pivotal roles in CCH. The most significant GO items were regulation of synapse structure or activity, protein serine/threonine kinase activity, transcription factor activity and binding. Reactome enrichment analysis showed that axon guidance and signaling by NGF and PDGF might also play a crucial role.

The aforementioned results suggest that energy metabolism, signal transmission, dopaminergic synapse signaling, cell death and survival, and neurotrophic factors regulate the associated pathways in CCH-related VD.

### IPA of Neuron Death Induced by CCH

IPA software program can analyze the gene expression patterns using a build-in scientific literature based database (Yu et al., 2016). The application of IPA analysis helps to decipher related functions, diseases, networks and even upstream regulator, thus may provide some new knowledge about VD induced by CCH.

In particular, IPA revealed nine mRNAs in CCH tissues, which are involved in cell death and survival, apoptosis, necrosis, and neurological diseases. Additionally, the conservative score of Cyr61 was the highest, it is similar to humans. The present results revealed downregulation of Cyr61 mRNA and protein in CCH. Cyr61 is associated with cell proliferation, chemotaxis, angiogenesis, and cell adhesion. Cyr61 appears to play a role in wound healing, where it is upregulated in skin fibroblasts, the expression of a number of genes involved in angiogenesis and matrix remodeling. Cyr61 has been reported to participate in multiple functions, such as migration, proliferation, apoptosis in non-CNS organs (Jun and Lau, 2011). Moreover, Cyr61 is suggested to play an important role in the modulation of inflammatory cytokines and chemokines production (Kular et al., 2011). The expression and function of Cyr61 in the CNS remains poorly understood (Jones and Bouvier, 2014). And Atf3 is one of the most critical genes for ROS-induced stress responses, upregulates p53 expression and induces apoptosis in cells (Zhao et al., 2016). Ifit1 is a negative regulator of the inflammatory gene program (John et al., 2018). But little is known about the role of them in neurological diseases, further investigation is needed.

### Perspectives of Transcriptome Study in CCH and Significance of Clinical Transformation

The study of the genetics and epigenetics of neurological diseases both in mammalian model organisms and in humans is beginning to focus on genetic variants, which exist outside protein-coding genes, as potential drivers of pathogenesis. The conventional view of gene-regulation has focused on protein-coding genes until the discovery of numerous non-coding RNAs, including lncRNAs and circRNAs. Mapping studies, combined with whole-genome sequencing data using CCH rat models, have begun to provide evidence for the genetic basis of inheritance of traits (such as VD), which are exerted by non-protein-coding elements. A database of the transcriptome levels of differentially expressed RNAs and the co-expression networks and biological functions were built at the experimental animal level. The results in the present study should set a stage for inquiry of mRNAs, circRNAs, lncRNAs as candidates regulating the etiology of CCH. Validation of potential transcriptome biomarkers will provide experimental basis for further potential clinical targets of CCH related VD.

However, there are several limitations to this research which need to be considered. First, more time points of experimental groups are required in further investigation. Second, it is important to use as many animal models of CCH-induced VD and methods for evaluating cognitive function as possible to avoid problems associated with bias. Moreover, further experimental verification must be acquired with regards to analysis and compare to specimens such as cerebrospinal fluid/blood in patients in the next phase of our study.

## Conclusion

The present study elucidated WMD and different ways of cortical neuronal death were implicated in CCH, and thus resulted in cognitive impairment. The expression profiles and networks of mRNA–circRNA–miRNA and lncRNA–mRNA were profiled, and potential bioinformatic analysis were annotated. The study indicates that Cyr61 may be a key factor for cortical neuronal death in CCH. We plan to set up a database for further research, and the present results would inspire neuroscientific researchers to study the role of transcriptomic events in CCH, provide a fundamental basis including candidate biomarkers and therapeutic targets for the further clinical application.

## Ethics Statement

All experiments were conducted in accordance with National Institutes of Health guide for the care and use of laboratory animals. The protocol was approved by Laboratory Animal Ethics Committee of Jinan University.

## Author Contributions

WL and DW wrote the manuscript. WL, DW, JL, XX, and KS carried out the experiments. WL, XX, and JL contributed to imaging experiments. XX and KS contributed to Morris water maze task. WL and DW contributed to sample collection. XX and KS contributed to neuropathological experiments. WL, DW, JL, XX, and KS contributed to data analyses. LH designed the study and revised the manuscript.

## Conflict of Interest Statement

The authors declare that the research was conducted in the absence of any commercial or financial relationships that could be construed as a potential conflict of interest.

## Footnotes

1 http://www.lncipedia.org

2 http://www.geneontology.org

3 http://www.ingenuity.com

4 https://blast.ncbi.nlm.nih.gov/Blast.cgi

## Supplementary Material

FIGURE S1 Full western blots from *in vivo* experiments.

## Abbreviations

Apold1: apolipoprotein L domain containing 1
Atf3: activating transcription factor 3
Cyr61: cysteine-rich angiogenic inducer 61 or CCN family member 1 (CCN1)
HIF1 α: hypoxia-inducible factor 1 α
Iift1: interferon-induced protein with tetratricopeptide repeats 1
Iift3: interferon-induced protein with tetratricopeptide repeats 3
MLKL: mixed lineage kinase domain-like protein
NR4A2: NR4A orphan nuclear receptor family member 2
PDE4C: phosphodiesterase 4C
pRIPK3: phosphorylated receptor-interactive protein kinase 1 protein
RIPK1: receptor-interactive protein kinase 1
RIPK3: receptor-interactive protein kinase 3 protein
TNF α: tumor necrosis factor α
ZO-1: Zonula occludens-1.
