# Corrigendum: Comprehensive Evaluation of White Matter Damage and Neuron Death and Whole-Transcriptome Analysis of Rats With Chronic Cerebral Hypoperfusion

**DOI:** 10.3389/fncel.2020.616236

**Published:** 2020-12-11

**Authors:** Wenxian Li, Di Wei, Jianye Liang, Xiaomei Xie, Kangping Song, Li'an Huang

**Affiliations:** ^1^Department of Neurology, The First Affiliated Hospital, Jinan University, Guangzhou, China; ^2^Department of Neurology, The Second Affiliated Hospital, Xi'an Jiaotong University, Xi'an, China; ^3^Department of Urology, Xijing Hospital, The Fourth Military Medical University, Xi'an, China; ^4^Medical Imaging Center, The First Affiliated Hospital, Jinan University, Guangzhou, China

**Keywords:** chronic cerebral hypoperfusion, white matter damage, neuron death, whole-transcriptome, vascular dementia

In the original article, there was a mistake in ^*^^*^Figure 2C2^*^^*^ as published. ^**^The third image in original **Figure 2C2** was incorrect^**^. The corrected ^**^Figure 2C2^**^ appears below.

**Figure 2:**
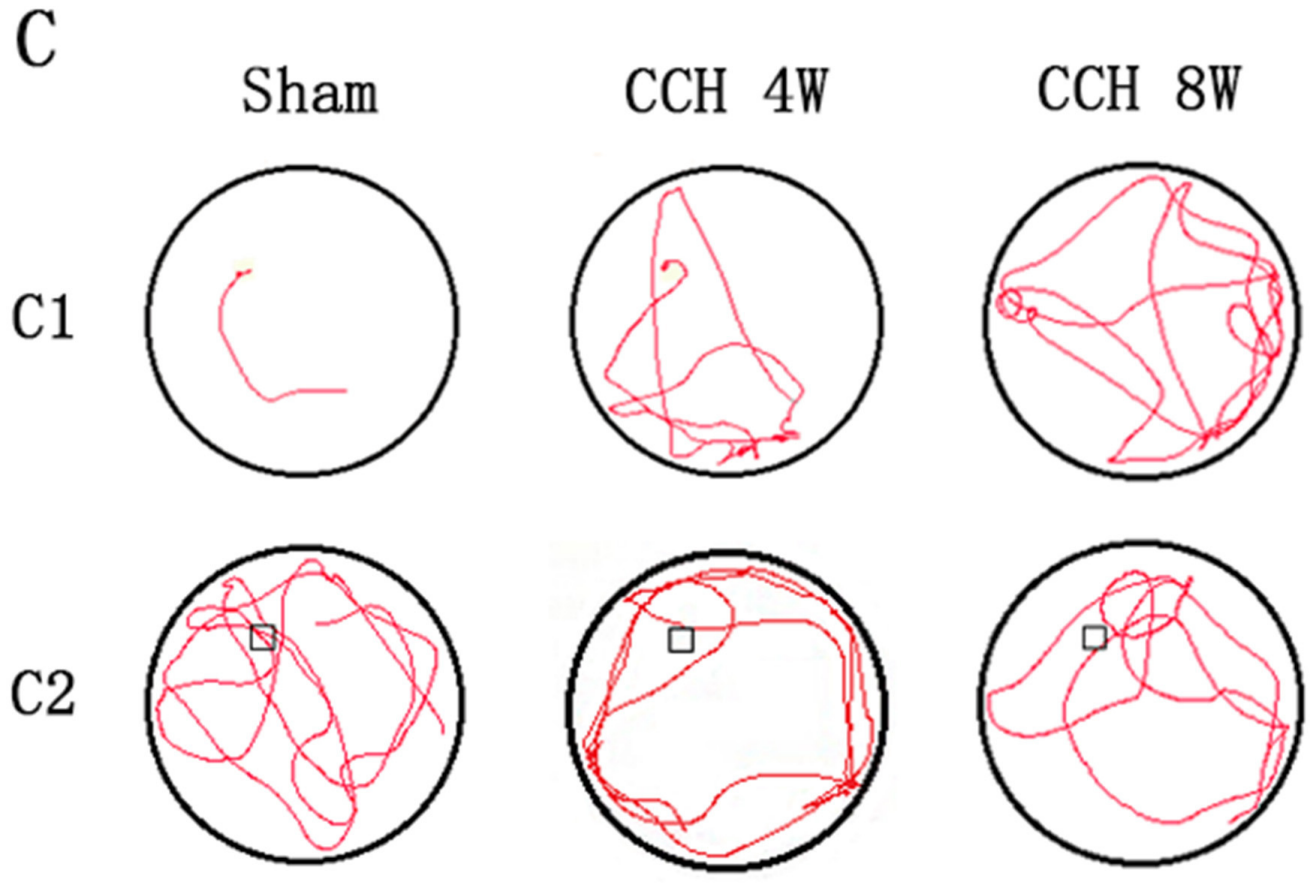


The authors apologize for this error and state that this does not change the scientific conclusions of the article in any way. The original article has been updated.

