# Supplementary figures and images for: Comprehensive Evaluation of White Matter Damage and Neuron Death and Whole-Transcriptome Analysis of Rats With Chronic Cerebral Hypoperfusion
Source: Front Cell Neurosci. 2019 Jul 17;13:310. doi: 10.3389/fncel.2019.00310 (PMC6653095; doi:10.3389/fncel.2019.00310)

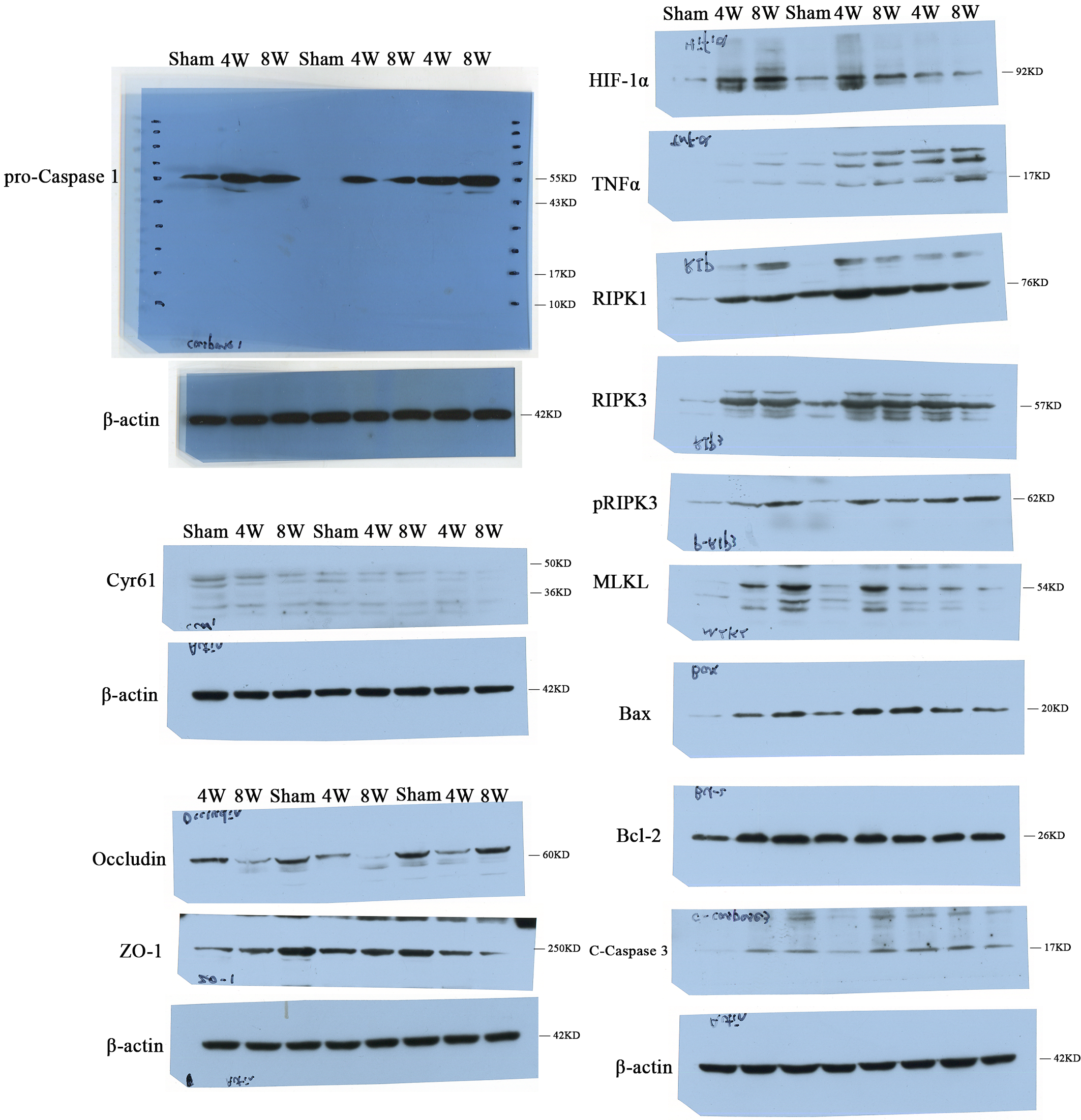

Supplement: FIGURE S1 — Full western blots from in vivo experiments. [file Image_1.TIF]
